# Taxonomic description of a novel genus, Parajatrophihabitans gen. nov., in the family Jatrophihabitantaceae

**DOI:** 10.1099/ijsem.0.007176

**Published:** 2026-05-27

**Authors:** Imen Nouioui, Sarah Kirstein, Gabriele Pötter, Marlen Jando, Juan Pablo Gomez Escribano, Meina Neumann-Schaal, Mathias Müsken, Cathrin Spröer, Boyke Bunk, Yvonne Mast

**Affiliations:** 1 Leibniz-Institute DSMZ – German Collection of Microorganisms and Cell Cultures, Inhoffenstraße 7B, 38124 Braunschweig, Germany; 2 Braunschweig Integrated Centre of Systems Biology (BRICS), Rebenring 56, 38106 Braunschweig, Germany; 3 Central Facility for Microscopy, Helmholtz Centre for Infection Research (HZI), 38124 Braunschweig, Germany; 4 Technische Universität Braunschweig, Institut für Mikrobiologie, Rebenring 56, 38106 Braunschweig, Germany

**Keywords:** actinomycetes, diversity, microbial ecology, systematics, taxonomy

## Abstract

Strain DSM 45814^T^, isolated from forest soil in Canada, was subjected to a polyphasic taxonomic study and genome mining for plant growth-promoting genes. The strain had a 16S rRNA gene sequence similarity of 97.1% with that of *Jatrophihabitans telluris* N237^T^ and 94.8–96.8% similarity with other validly named *Jatrophihabitans* species. Average nucleotide identity and digital DNA–DNA hybridization values between DSM 45814^T^ and its closely related *Jatrophihabitans* strains were below the established prokaryotic species demarcation. In the genome-based phylogeny, strain 45814^T^ was divergent from the *Jatrophihabitans* cluster and was loosely associated with the family *Geodermatophilaceae*. The average amino acid identity (AAI) values between DSM 45814^T^ and type (63.5–71.6%) and non-type (63.0–71.6%) strains of *Jatrophihabitantaceae*, as well as members of *Geodermatophilaceae* (58.6–59.2%), fell within the defined AAI range of 65–72% and below the recently established cut-off point of 74–76% for genus demarcation. The percentage of conserved proteins (POCP) between strain DSM 45814^T^ and the type strains of *Jatrophihabitantaceae* and *Geodermatophilaceae* was below the defined threshold of 50% for genus demarcation, excluding *J. telluris*, which had a POCP of 55%. Strain DSM 45814^T^ displayed cocci-to-cuboid cells with a flagellum and a G+C content (63.4 mol%) that distinguished it from *Jatrophihabitans* and *Geodermatophilaceae* strains, but it had chemotaxonomic features closer to those of *Jatrophihabitantaceae* than *Geodermatophilaceae*. The strain appeared to have ecological potential based on a genome mining approach. Based on these results, strain DSM 45814^T^ (=899^T^=LMG 34134^T^) represents a novel genus within the family *Jatrophihabitantaceae*, for which the name *Parajatrophihabitans canadensis* gen. nov. sp. nov. is proposed.

## Introduction

*Actinomycetota* is one of the largest and most diverse phyla within the domain *Bacteria*, encompassing strains with a wide range of lifestyles, e.g. saprophytic, pathogenic or plant symbiotic [1]. Actinomycetes are ubiquitous across diverse ecosystems, such as soil, freshwater, marine environments, deserts, Antarctica and other extreme environments, where they play an important role in global carbon cycling. They enhance soil fertility through the mineralization of organic matter and immobilization of mineral nutrients [2]. These micro-organisms are prolific producers of natural products (NPs) with biotechnological, pharmaceutical and agricultural applications, including antibiotics, antifungal, immunosuppressive and anticancer agents [3,7].

The promising potential of these micro-organism calls for further exploration of their diversity with particular focus on ‘rare *Actinobacteria*’ that are known for their ecological and agricultural potential but also as a rich source of novel NPs [8,10]. Rare taxa, defined as bacteria less frequently isolated under standard cultivation conditions, constitute ~10% of all isolated actinomycetes [11] and encompass more than 220 genera, with more than 50 taxa reported to produce around 2,500 bioactive compounds [9,10]. Despite this potential, many rare taxa remain poorly studied, and their functional applications are not well understood. One such example is the genus *Jatrophihabitans* of the family *Jatrophihabitantaceae*, and order *Jatrophihabitantales*. Currently, this genus comprises six validly named species, with *Jatrophihabitans endophyticus* as the type strain [12] (https://lpsn.dsmz.de/genus/jatrophihabitans). *Jatrophihabitans* strains have been isolated from diverse environments and geographical regions, including plants (*J. endophyticus* and *Jatrophihabitans huperziae*), rhizosphere (*Jatrophihabitans cynanchi*), soil (*Jatrophihabitans soli* and *Jatrophihabitans fulvus*) and sandstone (*Jatrophihabitans lederbergiae*), with samples originating from locations within Antarctica, China, Korea and Singapore. These bacteria are Gram-positive, mainly rod-shaped and non-spore-forming. Chemotaxonomically, they are characterized by the presence of *meso*-diaminopimelic acid (*meso*-DAP) in their cell-wall peptidoglycan; predominant menaquinones MK-9(H_4_), MK-9(H_6_) and MK-9(H_8_); diphosphatidylglycerol (DPG) as the major polar lipid; and *iso*-C_16 : 0_ as the dominant fatty acid [12,13].

Screening the DSMZ actinobacteria collection for rare taxa, strain DSM 45814^T^, originally deposited at the Leibniz Institute DSMZ-German Collection of Microorganisms and Cell Cultures (DSMZ) as a *Jatrophihabitans* strain, was selected for a polyphasic taxonomic study. The potential application of the strain was assessed based on *in silico* analyses. The results of this study provide insight into the plant growth-promoting features of the strain.

## Origin and growth properties

Strain DSM 45814^T^ (=899^T^=LMG 34134^T^) was isolated from forest soil collected in Canada (2010) and deposited at the DSMZ open culture collection in 2012 by Dr. Roland Wilhelm, University of British Columbia, Vancouver, Canada. For comparative purposes, *J*. *endophyticus* DSM 45627^T^, the type species of the genus, was included in this study. This reference strain was obtained from the DSMZ culture collection (https://www.dsmz.de/collection/catalogue).

DSM 45814^T^ is a slow-growing actinobacterium that requires 7–14 days of incubation at 28 °C to obtain an active culture. Therefore, a 10-day-old culture of DSM 45814^T^, grown on DSMZ 553 medium at 28 °C to a cell density equivalent to 5 on the McFarland scale, was used to determine the optimal growth condition, as well as biochemical and enzymatic properties. The growth ability of DSM 45814^T^ was assessed across various culture media, including Czapek peptone agar (DSMZ 83), International *Streptomyces* Project (ISP) media [ISP1 (DSMZ 1764), ISP2 (DSMZ 987), ISP3 (DSMZ 84), ISP4 (DSMZ 252), ISP5 (DSMZ 993), ISP6 (DSMZ 1269) and ISP7 (DSMZ 1619)], Glucose-Yeast extract-Malt extract (DSMZ 65), Trypticase Soy Agar (DSMZ 535), N-Z amine (DSMZ 554), GPHF (DSMZ 553) and R2A (DSMZ 830). In addition, the growth of the strain was evaluated across a wide range of temperatures (4 °C, 10 °C, 15 °C, 25 °C, 28 °C, 37 °C, 42 °C and 45 °C), pH levels (5.0, 5.5, 6.0, 6.5, 7.0, 7.5, 8.0, 8.5 and 9.0) and NaCl concentrations [2.5%, 5.0%, 7.5% and 10.0% (w/v)]. The ability of the strain to grow under anaerobic conditions was tested using anaerobic atmosphere-generating bags (AnaeroGen^™^ Compact, Thermo Scientific AN0020D). All tests listed above were performed in duplicate using a 10-day-old culture of the strain grown on DSMZ 553 medium at 28 °C. To determine cell morphology and dimensions via scanning electron microscopy (Merlin, Zeiss), bacterial colonies were fixed in 0.1 M EM-HEPES buffer (HEPES 0.1 M, 0.09 M sucrose, 10 mM CaCl2, 10 mM MgCl2 and pH 6.9), including 5% formaldehyde and 2% glutaraldehyde, and further processed as described in [14]. The flagella of strain DSM 45814^T^ were visualized and measured using negative staining and transmission electron microscopy (TEM), as described by Karagöz *et al*. [15].

The strain showed good growth on ISP2, ISP3 and DSMZ 553 agar media, and moderate growth on ISP1, ISP7, N-Z amine and GPHF agar media following 10 days of incubation at 28 °C. Colonies were beige to pale yellow across all media tested. The strain was able to grow within a temperature range of 10–28 °C and a pH range of 5.0 to 8.0 with optimal growth observed at 25°C–28 °C and pH 6.5 to 7.5. Poor growth was observed at 37 °C and in the presence of 2.5% NaCl. The strain was strictly aerobic.

**Fig. 1.**
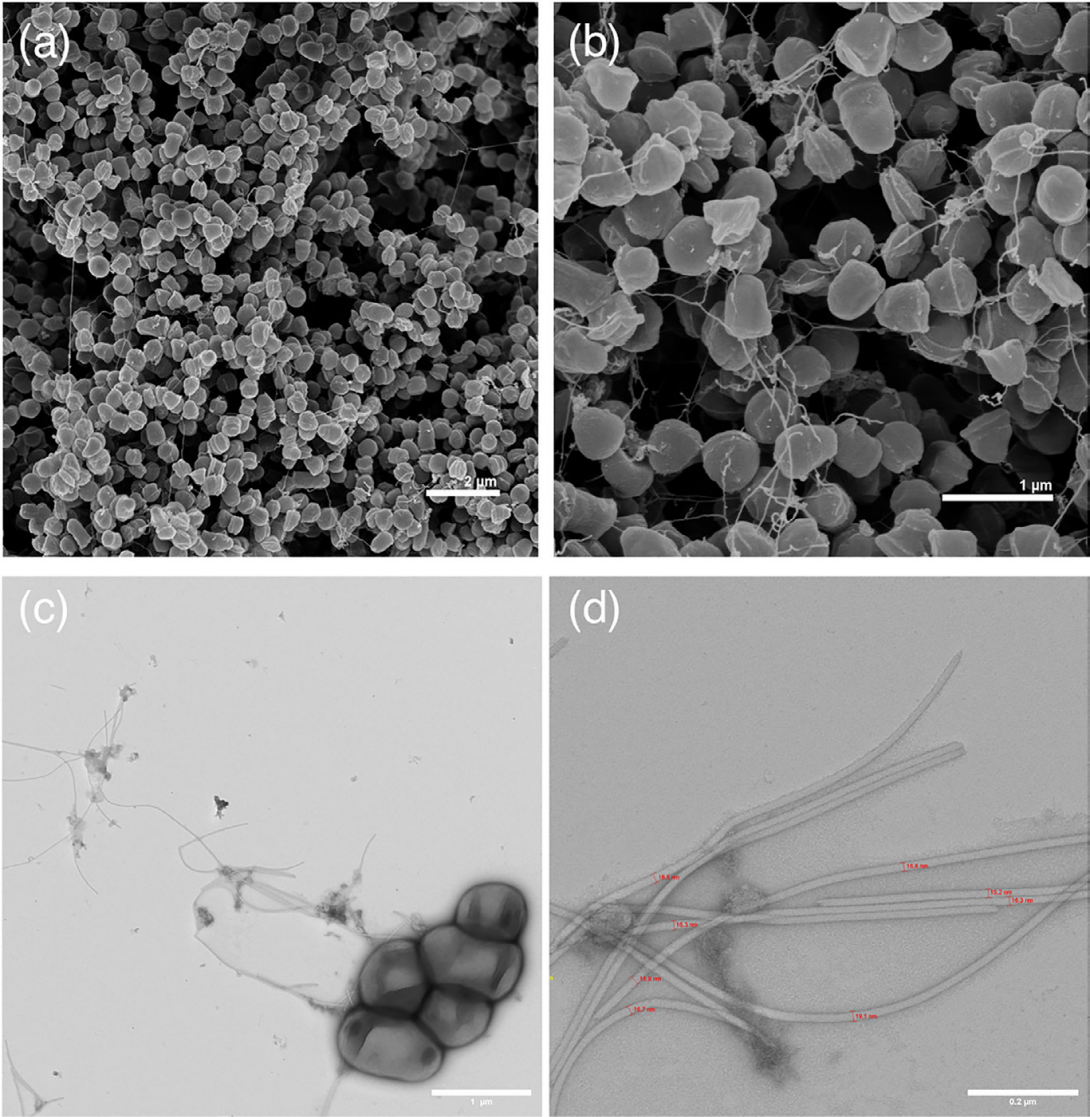
SEM (a, b) and TEM (c, d) micrographs of strain DSM 45814ᵀ showing clustered cocci to cuboid cells with a flagellum. Higher magnification reveals individual filament diameters measured near 15 to 19 nm. The strain was cultivated on medium DSMZ 553 at 28 °C for 10 days.

Microscopic examination revealed cocci-to-cuboid cells with a median size of 0.68 µm in length and 0.45 µm in width and flagella with 15.2–19.1 nm in diameter. The cellular morphology of strain DSM 45814^T^ distinguished it from *Jatrophihabitans* species, which are characterized by short rod-shaped cells (Fig. 1).

## Biochemical, enzymatic and chemotaxonomic characterizations

The biochemical and enzymatic profiles of strain DSM 45814^T^ were determined using API 50CH, API 20 NE and API ZYM kits following the manufacturer’s instructions (bioMérieux, France). The reference strain *J. endophyticus* DSM 45627^T^ was included for comparative analysis in all tests. All assays were performed in triplicate. Biomasses for biochemical and chemotaxonomic analyses were harvested from a 10-day-old culture of the strain grown on DSMZ 553 medium at 28 °C, washed three times with saline solution (0.9% NaCl) and freeze-dried. Chemotaxonomic markers of the strain, including whole-cell sugars [16,17], diaminopimelic acid (DAP) isomers [18], polar lipids [19,20], quinones [21] and fatty acids [22,23], were determined. Wet biomass was used for fatty acids and menaquinone analyses.

The biochemical and enzymatic analysis revealed that strain DSM 45814^T^ was able to metabolize d-cellobiose, produce *β*-galactosidase and reduce potassium tellurite, unlike *J. endophyticus*.

Whole-cell hydrolysate analysis revealed the presence of *meso-*DAP in the peptidoglycan of DSM 45814^T^ and a sugar profile rich in glucose, mannose, ribose, rhamnose and three unidentified sugars (Fig. S1, available in the online Supplementary Material). The fatty acid (>5%) of the strain consisted of *iso*-C_16 : 0_, C_16 : 0_, *anteiso*-C_17 : 0_, 10-methyl-C_17 : 0_ and 10-methyl-C_18 : 0_ (Table 1). Menaquinone analysis revealed MK-9(H_4_) as the predominant quinone (91.2%). These chemotaxonomic features were also found for the type strains of the families *Jatrophihabitantaceae* and *Geodermatophilaceae*, as shown in Table 1. Polar lipid analysis identified DPG, phosphatidylinositol (PI), glycophosphatidylinositol (GPI), glycophospholipid (GPL) and unidentified lipids (Ls) as polar lipids of DSM 45814^T^. This lipid profile was consistent with that of *Jatrophihabitantaceae* strains but differed from those observed for *Geodermatophilaceae* type strains (Table 1, Figs S1 and 2).

**Table 1.** Morphological, phenotypic, genetic and genomic features of strain DSM 45814^T^ and the type strains of the families *Jatrophihabitantaceae* and *Geodermatophilaceae*

	*Jatrophihabitantaceae*								*Geodermatophilaceae*							
	**DSM 45814^T^**	***J. endophyticus***	***J. cynanchi***	***J. fulvus***	***J. soli***	***J.*** ***lederbergiae***	***J. huperziae***	***J. telluris***	***G. obscurus***	***G. telluris***	***Klenkia marina***	***Modestobacter multiseptatus***	***Modestobacter muralis***	***Goekera deserti***	***Trujillonella endophytica***	***Blastococcus aggregatus***
Source	Soil	Stem tissue	Rhizosphere soil	Grass soil	Soil	Sandstone	Plant	Sediment soil	Soil	Sand	Marine sediment	Soil	Sandstone	Moss-dominated soil crust	Plant	Water
Cell morphology and size	Beige to pale yellow, cocci-to-cuboid cells	White and short rods	Pale yellow short rods	Yellow, short rods	White short rods	White beige, short rods	Pale orange, short rods	Pale yellow	Black cocci to cuboid	Black cocci to cuboids	Pink rod-shaped	Rod or cocci	Short rods and cocci	Cocci to rod-shaped	Cell in pairs or tetrads	Rod-shaped cells
Cell size	0.3–0.6 µm×0.4–1.3 µm	1.0–1.4 µm×0.4–0.63 µm	1.0–1.2×0.5–0.7 µm	1.0–1.3 µm×0.4–0.6 µm	0.5–0.6 µm×0.9–1.4 µm	–	0.5–0.7×1.0– 1.1 µm	0.4–0.5×1.0–1.1 µm	0.5–2.0	–	–	1.0–2.8×1.0–3.0 µm	–	–	–	Rods or ellipsoid (1.2– 1.5×1.5–3.0 µm); vibrioid (0.3–1.5×0.4–3.0 µm)
Motility	Single flagellum with 15.2–19.1 nm in diameter	–	Single flagellum	Non-flagellated	Non-flagellated	–	Non-flagellated	Non-flagellated	Motile zoospores	Motile zoospores	Motile	Motile	Motile	Motile	Non-motile	Motile and non-motile rods
Physiology	Strict aerobic	Aerobic	Aerobic	Aerobic	Aerobic	Aerobic	Aerobic	Aerobic	Aerobic	Aerobic	Aerobic	Aerobic	Aerobic	Aerobic	Aerobic	–
**Chemota-** **xonomic features**																
Quinone	MK-9(H_4_)	MK-9(H_4_), MK-9(H_6_)	MK-9(H_4_) and MK-9(H_6_)	MK-9(H_4_), MK-9(H_6_) with trace MK-9(H_8_)	MK-9(H_4_), MK-9(H_6_)	–	MK-9(H_4_), MK-9(H_6_), MK-9(H_8_)	MK-9(H_4_), MK-9(H_6_), MK-9(H_8_)	MK-9(H_4_), MK-9(H_2_), two MK	MK-9(H_4_)	MK-9(H_4_) and MK-9(H_0_)	MK-9(H%), but MK-8(H%) and MK-9(H')	MK-9(H_4_)	MK-9(H_4_), with minor of MK-8(H_4_)	MK-9(H_4_), with MK-8 and MK-9(H_6_) as minor components	MK-9(H_4_); MK-9
Polar lipids	DPG, PI, GPI, GPL, Ls	DPG, PL, AL, GL	DPG, PI, PIM, AGPL	DPG, PI, GL, PL, PAL	DPG, PI, Al, GL	DPG, AL, PME, GAPI, GPL, PI, PLs, PAL	DPG, PL, AL	DPG, PI, PM, PL, APLAL, GPL, GL, L	DPG, PE, PG, PC, PI	DPG, PE, PG, PC, PI, PN	DPG; PE; OH^-^PE (trace); PI; GPI, AL	DPG, PE, PI; PG	–	DPG, PE, PG, PI and PIM, as well as small amounts of APL	DPG, PC, PE, PI, PG, PIM	DPG, PG, PI, PE, PL1-2
Peptid- oglycan type	*meso*-DAP	*meso*-DAP	*meso*-DAP	–	*meso*-DAP	*meso*-DAP	*meso*-DAP	*meso*-DAP	*meso*-DAP	*meso*-DAP	*meso*-DAP	*meso*-DAP	*meso*-DAP	*meso*-DAP	*meso*-DAP	*meso*-DAP
Major fatty acids	*iso*-C_16 : 0_, C_16 : 0_, *anteiso*-C_17 : 0_, 10-methyl-C_17:0_ and 10-methyl-C_18 : 0_	*iso*-C_16 : 0_, C_18 : 1_ ω9c, *anteiso*-C_17 : 0_, C_17 : 1_ ω8c	*iso*-C_16 : 0_	*iso*-C_16 : 0_, C_18 : 1_ ω9c, C_17 : 1_ v8c	*iso*-C_16 : 0_	*iso*-C_16 : 1_ cis9, *iso*-C_16 : 0_, C_17 : 1_ cis9, 10-methyl-C_17 : 0_, C_18 : 1_ cis9, TBSA 10-methyl-C_18 : 0_	*iso*-C_16 : 0_, *anteiso*-C_17 : 0_, *anteiso*-C_15 : 0_, *iso*-C_14 : 0_ and C_15 : 0_	*iso*-C_16 : 0_, C_17 : 1_ ω8c, lower amounts of C_18 : 1_ ω9c and C_17 : 0_	*iso*-C_15 : 0_, *iso*-C_16 : 0_, C_17 : 1_ ω8c	*iso*-C_15 : 0_, *iso*-C_16:0_	*iso*-C_16:0_, *iso*-C_15:0_	C_18 : 1_, *anteiso*-C_17 : 0_, *iso*-C_16:0_	*iso*-C_15 : 0_, *iso*-C_16:0_, C_16 :0_, C_17:1_, x9c, C_17 : 0_	C_18 : 1_* ω*9*c*, *iso*-C_16 : 0_, C_16 : 0_, *iso*-C_15 : 0_, C_16 : 1_* ω*7*c*	*iso*-C_16 : 0_, *iso*-C_15 : 0_, C_18 : 1_* ω*9c	*iso*-C_16 : 0_, *iso*-C_16 : 1_
Whole-cell sugars	Glucose, mannose, ribose, rhamnose	–	–	–	–	glu, rib, rham, with traces of man, galac	–	–	–	Galactose	Rhamnose, ribose, mannose, glucose and unidentified sugar	Glutamate, alanine and glycine, as well as galactose, glucose and ribose	Glucose, galactose and ribose	Arabinose, galactose, ribose and traces of glucose	Arabinose and galactose	–
**Genomic features**																
Size (Mb)	5.1	4.5	4.5	–	–	5.2	–	4.2	5.3	4.8	4.2	–	4.5	4.9	4.9	4.6
G+C content (mol%)	63.4	73.0	70.7	72.4	72.1	68.2	71.9	68.1	74.0	75.5	74.0	68–70	71.5	74.7	74.6	73.9
References	*	[15]	[59]	[16,37,40]	[16,61]	[53]	[16,62]	[16]	[63,65]	[66]	[64]	[67]	[68]	[69,70]	[70,71]	[72]

–, data not available.

*Present study.

## Molecular identification

### 16S rRNA gene-based identification

To determine the phylogenetic position of strain DSM 45814^T^, genomic DNA was extracted from a 10-day-old culture grown on DSMZ 553 medium at 28 °C and subjected to PCR amplification of the 16S rRNA gene [24]. The resulting 16S rRNA amplicon was sequenced using the Applied Biosystems (ABI) 96-capillary system, as described in Risdian *et al*. [25]. The 16S rRNA gene sequence of the closest neighbours, comprising the seven *Jatrophihabitans* species with valid names, and the pairwise 16S rRNA gene sequence similarity values were retrieved from the EzBioCloud server (https://www.ezbiocloud.net/) [26]. A neighbour-joining phylogenetic tree based on the 16S rRNA gene sequences was inferred via mega (Molecular Evolutionary Genetics Analysis) version 12 software, with 1,000 bootstrap replicates [27,30]. The authenticity of strain DSM 45814^T^ was confirmed by comparing the 16S rRNA gene sequence (>1,400 bp) of the strain derived from the PCR reaction with the corresponding sequence extracted from its whole-genome assembly using blastn on the NCBI server (https://blast.ncbi.nlm.nih.gov/Blast.cgi) [31,32]. Pairwise 16S rRNA gene sequence similarity between strain DSM 45814^T^ (1,524 bp) and the type strain of *Jatrophihabitans* species ranged from 95.2 to 97.1%. The closest relative was *Jatrophihabitans telluris* N237^T^ (97.1%), followed by *J. lederbergiae* DSM 44399^T^ (96.8%), *J. endophyticus* DSM 45627 (95.9%), *J. soli* KIS75-12 (95.5%), *J. huperziae* CPCC 204076 (95.3%) and *J. fulvus* PB158 (94.8%). All values were below the 98.65% threshold established for bacterial species demarcation [33].

These results were consistent with the phylogenetic position of strain DSM 45814^T^ within the radiation of the genus *Jatrophihabitans* in the maximum likelihood phylogenetic tree. However, the strain formed a poorly supported subcluster with *J. telluris* (Fig. S3). This single-gene phylogeny indicates that 16S rRNA gene resolution is insufficient to conclusively determine the taxonomic status of strain DSM 45814^T^, necessitating further taxogenomic analyses.

### Genome-based phylogeny and comparative genomic analyses

To elucidate the phylogenomic position and genomic characteristics of strain DSM 45814^T^, the genomic DNA was extracted and sequenced using the PacBio Sequel *II* platform (Pacific Biosciences, Menlo Park, CA, USA). SMRTbell^®^ template library was prepared using the SMRTbell^®^ prep kit 3.0 and according to the instructions from Pacific Biosciences, Menlo Park, CA, USA. Briefly, for the preparation of 10 kb libraries, 2 µg of genomic DNA was sheared using the Megaruptor^®^ 3 from Diagenode, Denville, NJ, USA, according to the manufacturer’s instructions. DNA was end-repaired and ligated to barcoded adapters applying components from the SMRTbell^®^ prep kit 3.0 from Pacific Biosciences, Menlo Park, CA, USA. Reactions were carried out according to the manufacturer’s instructions. Samples were pooled equimolarly. Conditions for annealing of sequencing primers and binding of polymerase to purified SMRTbell^®^ template were assessed according to the Sample Setup in SMRT^®^Link, Pacific Biosciences, Menlo Park, CA, USA. Libraries were sequenced on the Sequel IIe (Pacific Biosciences, Menlo Park, CA, USA), taking one 30 h movie per SMRT cell. Genome quality control was assessed using the LongQC v.1.2.0c software [34], and no error correction was applied. Long-read genome assembly was performed using the ‘Microbial Genome Analysis’ protocol included in SMRTLink version 11.1 using default parameters. One circular chromosomal contig of 5.1 Mbp was obtained and rotated to the *dnaA* gene. The genome of strain DSM 45814^T^ has been deposited in the NCBI GenBank database and automatically annotated via PGAP [35,37]. Whole genome-based phylogenetic analysis was conducted using the Type Strain Genome Server [TYGS (https://tygs.dsmz.de/)] [38,39]. To improve the resolution and capture the genetic diversity within the genus, 17 genomes of uncharacterized *Jatrophihabitans* strains available in GenBank were included in this study.

Digital DNA–DNA hybridization (dDDH) values between the whole-genome sequence of strain DSM 45814^T^ and its close phylogenomic neighbours were calculated via the TYGS web server using the recommended formula *d_4_* [30]. Average nucleotide identity (ANI) and average amino acid identity (AAI) between the strain DSM 45814^T^ and members of the families *Jatrophihabitantaceae* and *Geodermatophilaceae* were estimated using the ANI calculator and EzAAI (average amino acid sequence identity) tools available on the EzBioCloud server [40], respectively. An AAI-based dendrogram was constructed via the EzAAI pipeline [41]. Additionally, the percentage of conserved proteins (POCP) between pairs of genomes was calculated using the POCP-nf version '2.3.4' pipeline with default settings, according to the original method described in Qin *et al*. [42]. Protein alignments were performed with DIAMOND (e-value: 1e-5; sequence identity: 0.4; alignment length: 0.5) [42,43].

**Fig. 2.**
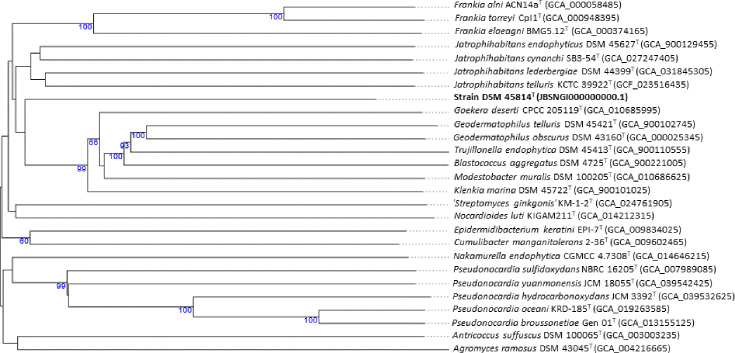
GBDP phylogenetic tree inferred from GBDP distances calculated from genome sequences showing the phylogenetic relationship of strain DSM 45814^T^ and its close relatives. The branch lengths are scaled in terms of GBDP distance formula *d_5_*. The numbers above branches are GBDP pseudo-bootstrap support values >60% from 100 replications.

Strain DSM 45814^T^ showed a genome size of 5.13 Mb, with an L50 of 1, 5,153 coding sequences and 49 RNAs. DNA base composition (G+C mol%) has been considered a genotypic marker for a standard description of bacterial taxa [44] and has been defined at 1% within the same species based on genomic data [45]. In this context, the strain had a G+C content of 63.4 mol%, which was well below the G+C content range for the genus *Jatrophihabitans* (68.3–71.2%) and the type species within the family *Geodermatophilaceae* (68.0–75.0%), indicating substantial divergence from the currently described species. Phylogenomic analysis revealed that strain DSM 45814^T^ formed a distinct and distant branch from the *Jatrophihabitans* clade, including *J. telluris*, and showed only loose association with a well-supported cluster of type species of the genera *Blastococcus*, *Geodermatophilus*, *Goekera*, *Klenkia*, *Modestobacter* and *Trujillonella* within the family *Geodermatophilaceae* (Fig. 2). The *Jatrophihabitans* clade comprised two subclusters each containing a pair of type strains: *J. endophyticus* and *J. cananchi* on the one hand, and *J. lederbergiae* and *J. telluris* on the other. After including 21 genomes of non-type strains of *Jatrophihabitans* in the phylogenomic tree (Fig. S4), all *Jatrophihabitans* strains formed a well-supported cluster in which *J. endophyticus* and *J. cynanchi* species were placed in one subcluster, while *J. lederbergiae* and *J. telluris* were grouped in another subcluster. However, strain DSM 45814^T^ was clustered within the family *Nocardiaceae*, forming a poorly supported subclade with the type strain of *Rhodococcus antarcticus* DSM 44784ᵀ (Fig. S4).

The clear phylogenomic separation of strain DSM 45814^T^ from the clade housing the *Jatrophihabitans* strains and its loose association with the *Geodermatophilaceae* or *Nocardiaceae* clusters required more in-depth taxogenomic investigation.

The dDDH values between the genome sequence of DSM 45814^T^ and type strains of the families *Jatrophihabitantaceae* and *Geodermatophilaceae* were below 21.1%, which was significantly lower than the defined threshold of 70% for prokaryotic species demarcation [46] (Table 2). The ANI values between strain DSM 45418^T^ and the type strains of *Jatrophihabitantaceae* and *Geodermatophilaceae* ranged from 71.5 to 73.5% and 69.7 to 70.4%, respectively, which were below the accepted thresholds of 95–96% for species and 73.1% for genus demarcation, respectively [47,49]. Notably, the ANI scores between strain DSM 45418^T^ and *J. lederbergiae* (73.5%) and *J. telluris* (73.0%) were at the borderline of the recently defined threshold of 73.1% for bacterial genus delineation [48] (Table 2).

**Table 2.** Genome-related indices (dDDH, ANI, AAI and POCP) between the genome sequence of strain DSM 45814^T^ and the type strains of the families *Jatrophihabitantaceae* and *Geodermatophilaceae*

dDDH	ANI	AAI	POCP
***Jatrophihabitantaceae***
*J. cynanchi*	20.8	71.3	64.3	47.3
*J. endophyticus*	19.8	71.5	63.5	46.2
*J. lederbergiae*	20.3	73.5	70.9	46.2
*J. telluris*	20.4	73.0	71.6	55.8
***Geodermatophilaceae***
*G. obscurus*	20.2	70.4	59.1	36.4
*G. telluris*	20.5	70.2	59.2	39.0
*K. marina*	21.0	69.7	58.8	39.4
*G. deserti*	21.0	69.9	59.0	38.6
*T. endophytica*	20.2	70.0	58.6	42.4
*B. aggregatus*	19.0	70.1	58.8	38.2
*M. muralis*	20.8	70.4	59.0	37.1
*M. multiseptatus*	20.5	69.7	59.0	42.4

The genome sequences of *J. huperziae*,* J. fulvus* and *J. soli* are not available.

**Fig. 3.**
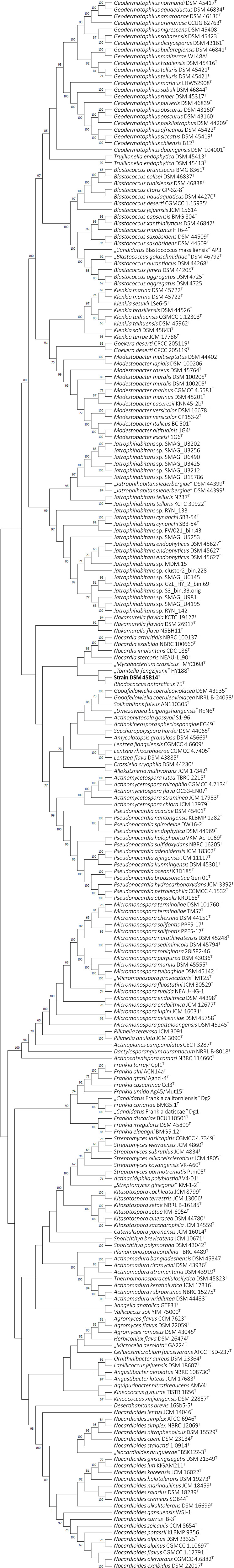
GBDP phylogenetic tree inferred from GBDP distances calculated from genome sequences showing the phylogenetic relatedness of strain DSM 45814^T^ to *Jatrophihabitantaceae* (including 21 non-type strains) and *Geodermatophilaceae* strains. The branch lengths are scaled in terms of GBDP distance formula *d_5_*. The numbers above branches are GBDP pseudo-bootstrap support values >60% from 100 replications.

The AAI values between the genome sequences of strain DSM 45814^T^ and type strains of *Jatrophihabitantaceae* (63.5–71.6%) and *Geodermatophilaceae* (58.6–59.2%), as well as non-type strains of *Jatrophihabitantaceae* (63.0–71.6%), were within the defined AAI range of 65–72% [50] and below the recently established cut-off point of 74–76% for genus demarcation [51,52] (Tables 2 and S1). In the AAI dendrogram, *Jatrophihabitans* strains were grouped into a cluster in which strain DSM 45814^T^ formed a subcluster with *J. telluris*, closely related to a cluster containing *J. lederbergiae* DSM 44399^T^ and six non-type strains of *Jatrophihabitans* (Fig. 3). All these strains were placed next to the cluster housing the type species of the genus *Jatrophihabitans*. The closeness of strain DSM 45814^T^ to *J. telluris* in the AAI dendrogram was not consistent with the GBDP trees, in which strain DSM 45814^T^ appeared in a separate branch, diverging from the genus *Jatrophihabitans* (Figs 2 and S4). The AAI dendrogram is based on overall similarity and does not represent an evolutionary model, which explains these discrepancies regarding the position of strain DSM 45814^T^. The divergence of this later from the genus *Jatrophihabitans* in both GBDP trees (Figs2  3), along with its loose association with *Geodermatophilaceae* and *Rhodococcus*, may be due to insufficient phylogenetic signal for confident placement, as the strain appears to be close to multiple groups. The distribution of *Jatrophihabitans* strains into two subclusters called for a taxonomic revision of this taxon based on POCP. The cluster of *Jatrophihabitans sensu stricto* (comprising the type species) exhibited POCP values above the defined threshold of 50% for genus delineation [42], while it had POCP values of 47.1 and 52.4% (>50%) with *J. lederbergiae* DSM 44399^T^ and *J. telluris* N237^T^*,* respectively (Table S1), though these type strains were placed in a distinct subcluster from *Jatrophihabitans sensu stricto* (Figs 3 and S4). *J. telluris* N237^T^ and *J. lederbergiae* DSM 44399^T^ had POCP values above 50% (except strain SMAG_U6490) when compared with the six non-type strains of *Jatrophihabitans* shown in the AAI dendrogram (Fig. 3). The POCP values between the type strains of *J. cynanchi*, *J. endophyticus*, *J. lederbergiae* and *J. telluris* ranged from 46.4% to 54.0%. The low POCP values (<50%) between *J. lederbergiae* DSM and *J. cynanchi* species (46.6%) and *J. endophyticus* (47.1%) questioned the assignment of the species *J. lederbergiae* to the genus *Jatrophihabitans*. The POCP data for *J. lederbergiae* species [POCP <50% with *J. cynanchi* (46.6%) and *J. endophyticus* (47.1%); POCP >50% with *J. telluris*] were not in concordance with the phylogenomic position of *J. lederbergiae* within the radiation of the genus *Jatrophihabitans*, as shown in Fig. 2 and in Nouioui *et al*. [53]. Previous studies have shown that the 50% POCP should not be used as a strict boundary for genus demarcation, as certain inconsistencies with AAI boundaries and/or monophyly have been observed [54,55]. Furthermore, *J. lederbergiae* shared several morphological, chemotaxonomic and biochemical features with the other validly named species *Jatrophihabitans* (Table 1) [53]. Consequently, no taxonomic conclusions can be drawn regarding the status of the species *J. lederbergiae* due to the limited number of genomes and species within this genus.

Furthermore, the POCP score between strain DSM 45814^T^ and the type strain of *Jatrophihabitantaceae* and *Geodermatophilaceae* (excluding *J. telluris*, which showed 55.8%) was below the 50% threshold for genus demarcation. The POCP values between DSM 45814^T^ and the non-type strain of *Jatrophihabitans* were also below 50%. These results were coherent with the distinct phylogenomic position of strain DSM 45814^T^ (Figs 2 and S4), though its association with the family *Jatrophihabitantaceae* and *Geodermatophilaceae* was poorly supported. Seven out of 17 non-type strains of *Jatrophihabitans* exhibited POCP values (50.4–60.3%) greater than 50% with the type species of the genus *J. endophyticus* (Tables 2 and S1).

The genome-related indices, including ANI (<73.5%), AAI (<72%) and POCP (<50%) between strain DSM 45814^T^ and the type species of *Jatrophihabitantaceae* (except *J. telluris*) and *Geodermatophilaceae* families, were all in concordance with the consistent phylogenetic divergence of the strain from these taxa (Figs2  3) and supported its classification to a novel genus, for which the name *Parajatrophihabitans canadensis* gen. nov. sp. nov. is proposed. The phenotypic, chemotaxonomic and biochemical features of the strain are more similar to those of members of the family *Jatrophihabitantaceae* than to those of *Geodermatophilaceae*. This taxonomic placement is supported not only by 16S rRNA gene-based phylogeny and genomic data (ANI, AAI and POCP) as outlined above, but also by its morphological, chemotaxonomic and genetic traits. Strain DSM 45814^T^ can be distinguished from validly named *Jatrophihabitans* species by its cocci-to-cuboid cell structure (a median size of 0.6 µm length and 0.4–1.3 µm width), a flagellum measuring 15.2–19.1 nm in diameter and a DNA G+C content of 63.4 mol%. In contrast, *Jatrophihabitans* strains have short rod cells (0.4–1.4 µm×0.4–1.4 µm), a variable presence of a single flagellum (no details on diameter are available) and a higher DNA G+C content ranging from 68.1% to 73.0%. These results support the assignment of the proposed novel genus to the family *Jatrophihabitantaceae*.

Strain DSM 45814^T^ and the type strain of *J. telluris* N237^T^ had 16S rRNA gene sequence similarity (97.1%), AAI (71.6%), ANI (73.0%) and POCP (55.8%) values that were slightly above the genus demarcation thresholds compared to the other type strains listed above. Genomic data support the transfer of *J. telluris* to *Parajatrophihabitans* gen. nov.; however, this is not consistent with its phylogenetic position in the 16S rRNA gene and genome-based trees (Figs 2, S3 and S4), where it was well inserted within the evolutionary radiation of the genus *Jatrophihabitans*, unlike strain DSM 45814^T^. Pauvert *et al*. [55] showed that the POCPu cutoff (using unique matches) for genus demarcation can vary from 48.8% to 63.1%, depending on the families within the phylum *Actinomycetota*. In this context, the POCP cutoff for the family *Jatrophihabitantaceae* can be estimated later when the diversity of this taxon expands with the inclusion of additional species.

### Potential application of strain DSM 45814^T^

To evaluate the ecological potential of strain DSM 45814^T^, its genome was screened for plant growth-promoting genes using the PGPT-Pred tool, available on the PLaBAse platform (https://plabase.cs.uni-tuebingen.de/pb/plabase.php) [56,58]. The analysis employed a combined blastp+hmmer approach under Krona strict mode against the PGPT ontology.

Genome mining for PGP-associated genes of strain DSM 45814^T^ revealed a broad range of gene clusters linked to plant-associated functions, such as plant colonization (26%), competitive exclusion (17%), phytohormone production (12%), bioremediation (10%) and stress biocontrol (21%). The presence of genes involved in iron and nitrogen acquisition, phosphate and potassium solubilization and sulphur assimilation suggests the potential of the strain to enhance soil fertility (Table S2). Moreover, the genomic data indicated that the strain had bioremediation potential since several gene clusters associated with detoxification of heavy metal (e.g. arsenic, bismuth, cadmium, chromate, cobalt, copper, gold, manganese, nickel, selenium, tellurium and zinc), herbicide (e.g. atrazine), hydrocarbon (e.g. naphthalene and toluene) and organic compounds (e.g. steroid and styrene) were present (Table S2). Furthermore, the strain harboured genes responsible for producing key phytohormones such as auxin, cytokinin, *γ*-aminobutyric acid and gibberellins. Several gene clusters encoding for compounds that promote germination were also identified, including H_2_S; lipoic acid; carotenoids; vitamins B1-2, B5-6, B9 and B12; terpenoids; ubiquinone; and volatiles. Additional gene clusters related to plant colonization and adaptation, such as those involved in cell wall degradation, root colonization, surface adhesion and host invasion, further reinforce the ecological potential of the strain. The presence of fitness-associated genes supports the potential of the strain to adapt and cope with different environmental conditions and makes it a good PGP candidate for bioremediation. More details are provided in Table S2. These results provide a foundation for further *in vitro* and *in planta* research studies to confirm the potential application of this strain.

## Conclusion

Phylogenomic, chemotaxonomic and phenotypic data (dDDH, ANI, AAI and POCP) consistently supported the assignment of strain DSM 45814^T^ to a novel genus within the family *Jatrophihabitantaceae*, for which the name *P. canadensis* gen. nov. sp. nov. is proposed. Genome mining for PGP genes highlights the ecological potential of the strain.

## Description of *Parajatrophihabitans* gen. nov.

*Parajatrophihabitans* (Pa. ra. ja.tro.phi.ha’bi.tans. Gr. prep. *para*, beside; N.L. masc. n. *Jatrophihabitans*, a bacterial genus name; N.L. masc. n. *Parajatrophihabitans*, resembling the genus *Jatrophihabitans*).

Gram-stain-positive and strictly aerobic actinobacterium with cocci-to-cuboid cells exhibiting a flagellum. Whole-cell hydrolysates were rich in glucose, mannose, ribose and rhamnose. The strain is characterized by the presence of *meso*-DAP in its peptidoglycan; DPG, PI, GPI, GPL and Ls as polar lipids; MK-9(H_4_) as predominant quinone; and *iso*-C_16 : 0_, C_16 : 0_, *anteiso*-C_17 : 0_, 10-methyl-C_17:0_ and 10-methyl-C_18 : 0_ as major fatty acids.

The type species is *P. canadensis*.

## Description *Parajatrophihabitans canadensis* sp. nov.

*Parajatrophihabitans canadensis* (ca.na.den’sis. N.L. masc. adj. *canadensis*, of or belonging to Canada, from where the organism was isolated).

Gram-stain-positive, strictly aerobic, cocci-to-cuboid cells with a median size of 0.68 µm in length and 0.45 µm in width, and flagellum with 15.2–19.1 nm in diameter. Beige to pale yellow colonies with creamy texture after incubation on DSMZ 553 agar medium for 10 days at 28 °C are developed. Optimal growth is observed at 25 and 28 °C in DSMZ 553 and DSMZ 830 media, pH 6.5–7.5 after 10 days of incubation at 28 °C. Metabolizes d-cellobiose, produces *β*-galactosidase, and reduces potassium tellurite. Whole-cell sugars are glucose, mannose, ribose, rhamnose and three unidentified sugars. The peptidoglycan contains *meso*-DAP. Polar lipid profile consists of DPG, PI, GPI, GPL and Ls. The predominant menaquinone is MK-9(H_4_), and major fatty acids (>5%) comprise *iso*-C_16 : 0_, C_16 : 0_, *anteiso*-C_17 : 0_, 10-methyl-C_17:0_ and 10-methyl-C_18 : 0_.

Strain DSM 45814^T^ (899^T^=LMG 34134^T^) was isolated from forest soil in the north of Prince George, Canada. The genome size is 5.1 Mb, and the G+C content is 63.4 mol%. The 16S rRNA gene and genome sequences have been deposited in GenBank under accession numbers PX702677 and JBSNGI000000000.1, respectively.

## Supplementary material

10.1099/ijsem.0.007176 Supplementary Material 1.

10.1099/ijsem.0.007176 Table S1.

## Abbreviations

AAI: average amino acid identity
ANI: average nucleotide identity
DAP: diaminopimelic acid
dDDH: digital DNA–DNA hybridization
DPG: diphosphatidylglycerol
DSMZ: Leibniz Institute DSMZ-German Collection of Microorganisms and Cell Cultures
ISP: International Streptomyces Project
Ls: lipids
*meso*-DAP: *meso*-diaminopimelic acid
NPs: natural products
PI: phosphatidylinositol
POCP: percentage of conserved proteins
SEM: Scanning electron microscopy
TEM: transmission electron microscopy
TYGS: Type Strain Genome Server
