# Supplementary material for: Taxonomic description of a novel genus, Parajatrophihabitans gen. nov., in the family Jatrophihabitantaceae
Source: Int J Syst Evol Microbiol. 2026 May 27;76(5):007176. doi: 10.1099/ijsem.0.007176 (PMC13215011; doi:10.1099/ijsem.0.007176)
Supplement: Supplementary Material 1. [file ijsem-76-07176-s001.pdf]

Taxonomic description of a novel genus, *Parajatrophihabitans* gen. nov., in the family *Jatrophihabitantaceae*

Imen Nouioui[1*], Gabriele Pötter[1], Marlen Jando[1], Juan Pablo Gomez-Escribano[1], Sarah Kirstein[1], Meina Neumann-Schaal[1,2], Mathias Müsken[3], Cathrin Spröer[1], Boyke Bunk[1], Yvonne Mast[1,2]

[1]Leibniz-Institut DSMZ – German Collection of Microorganisms and Cell Cultures, Inhoffenstraße 7B, 38124 Braunschweig, Germany.

[2] Braunschweig Integrated Centre of Systems Biology (BRICS), Rebenring 56, 38106 Braunschweig, Germany.

[3] Central Facility for Microscopy, Helmholtz Centre for Infection Research (HZI), 38124 Braunschweig, Germany

*Corresponding author: Imen Nouioui imen.nouioui@dsmz.de

Section: Actinobacteria

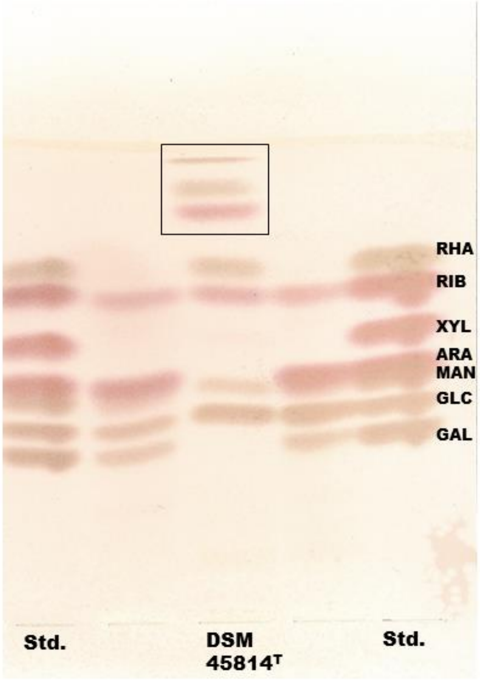

Figure S1. Thin-layer chromatogram plate of whole cell sugars extracted from strain DSM 45814^{T}. std, standard; RHA; rhamnose; RIB, ribose; XYL, xylose; ARA, arabinose; MAN, mannose; GLC, glucose; GAL, galactose.

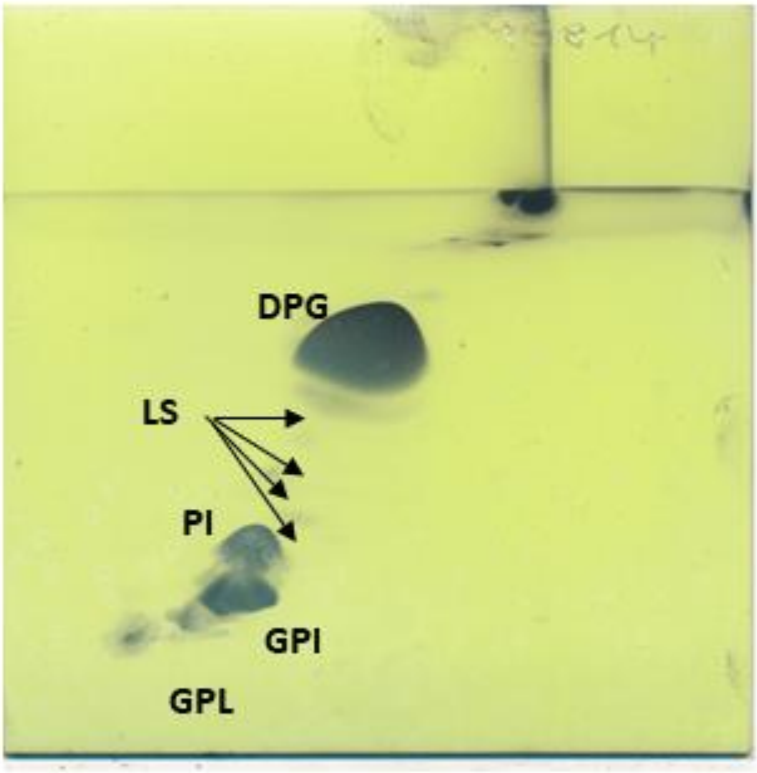

Figure S2. Two-dimensional layer chromatography plate of polar lipids extracted from strain DSM 45814^{T} stained with molybdatophosphoric acid (Sigma P1518). Key: DPG, diphosphatidylglycerol; PI, phosphatidylinositol; Ls, lipids; GPI, glycophosphatidylinositol;

GPL, glycophospholipid. Solvent 1: chloroform: methanol: distilled water (65:25:4 v/v/v); solvent 2: chloroform: glacial acetic acid: methanol: distilled water (80:12:15:4 v/v/v).

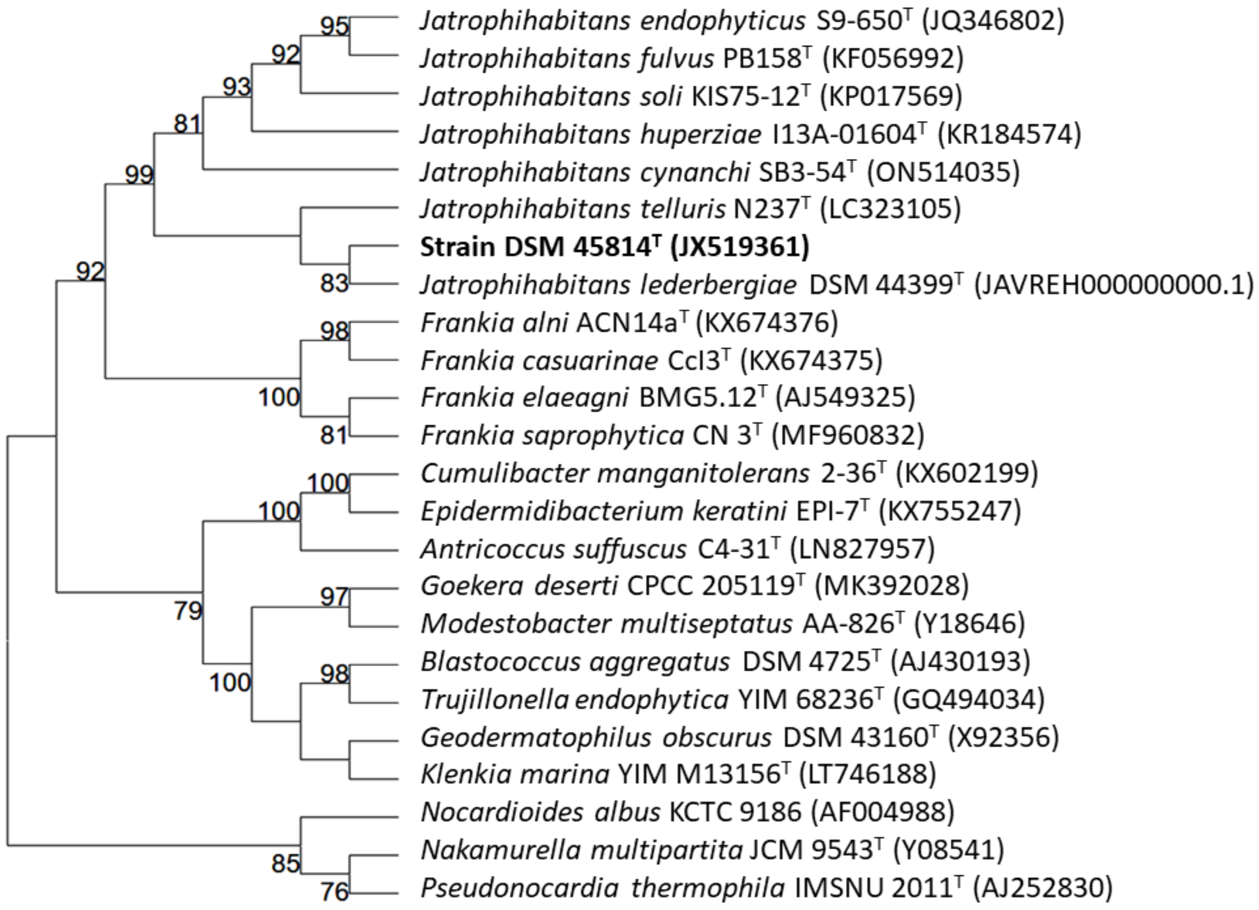

Figure S3. Neighbor-joining phylogenetic tree based on the 16S rRNA gene sequence showing the phylogenetic relationship of strain DSM 45814^{T} and its close neighbours.

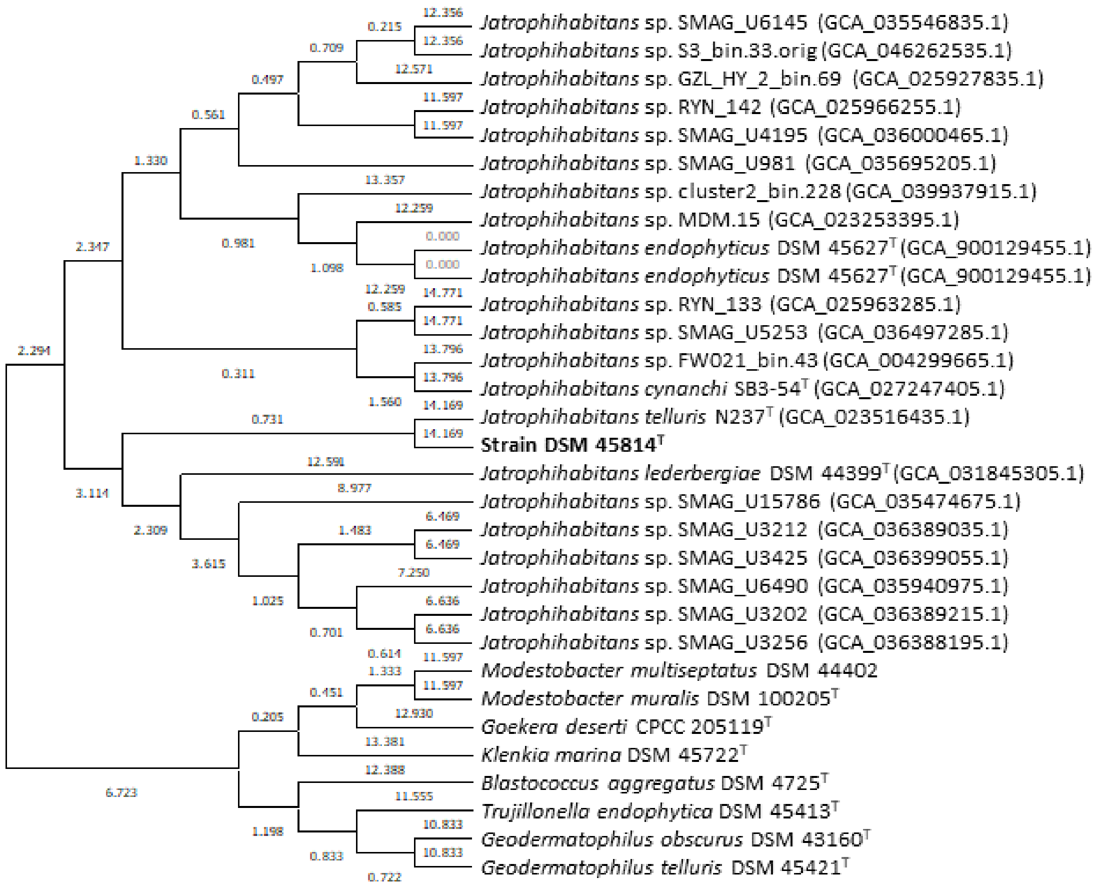

Figure S4. Dendrogram based on average amino acid identity between strain DSM 45814^{T} and its close neighbours.

Table S1. Percentage of Conserved Protein (PCOP) between strain DSM 41514^{T} and *Jatrophihabitans* including 21 non-type strains.

***Jatrophihabitans* strains**	**PCOP**		**Genome accession numbers**
	J. endophyticus	**DSM 41514^{T}**	
Jatrophihabitans cynanchi SB3-54^{T}	**54.0**	47.3	GCA_027247405.1
Jatrophihabitans huperziae CPCC 204076^{T}	-	-	-
Jatrophihabitans endophyticus DSM 45627^{T}	100	46.2	GCA_900129455.1
Jatrophihabitans fulvus PB158^{T}	-	-	-
Jatrophihabitans lederbergiae DSM 44399^{T}	47.1	46.2	GCA_031845305.1
Jatrophihabitans soli	-	-	-
Jatrophihabitans telluris N237 ^{T}	**52.4**	**55.8**	GCA_023516435.1
Jatrophihabitans sp. FW021_bin.43	45.8	37.9	GCA_004299665.1
Jatrophihabitans sp. MDM.15	47.4	29.9	GCA_023253395.1
Jatrophihabitans sp. GZL_HY_2_bin.69	49.7	41.5	GCA_025927835.1
Jatrophihabitans sp. RYN_133	**53.9**	47.5	GCA_025963285.1
Jatrophihabitans sp. RYN_142	50.4	42.1	GCA_025966255.1
Jatrophihabitans sp. SMAG_U15786	48.1	48.2	GCA_035474675.1
Jatrophihabitans sp. SMAG_U15786	46.7	37.6	GCA_035546835.1
Jatrophihabitans sp. SMAG_U981	45.3	36.0	GCA_035695205.1
Jatrophihabitans sp. SMAG_U6490	44.1	44.7	GCA_035940975.1

Jatrophihabitans sp. SMAG_U4195	**60.3**	48.4	GCA_036000465.1
Jatrophihabitans sp. SMAG_U3256	47.4	46.7	GCA_036388195.1
Jatrophihabitans sp. SMAG_U3212	48.0	47.6	GCA_036389035.1
Jatrophihabitans sp. SMAG_U3202	47.0	46.3	GCA_036389215.1
Jatrophihabitans sp. SMAG_U3425	49.7	47.7	GCA_036399055.1
Jatrophihabitans sp. SMAG_U5253	**53.3**	47.5	GCA_036497285.1
Jatrophihabitans sp. cluster2_bin.228	**53.3**	39.6	GCA_039937915.1
Jatrophihabitans sp S3_bin.33.orig	**56.9**	45.9	GCA_046262535.1

The PCOP in bold > 50%

Table S2.
